# Phase I dose-finding study of cabazitaxel administered weekly in patients with advanced solid tumours

**DOI:** 10.1186/1471-2407-13-460

**Published:** 2013-10-07

**Authors:** Pierre Fumoleau, Jose Manuel Trigo, Nicolas Isambert, Dorothée Sémiond, Sunil Gupta, Mario Campone

**Affiliations:** 1 Centre Georges-François Leclerc, 1 rue Professeur Marion, 21079 Dijon Cedex France; 2 Servicio de Oncología Médica, Hospital Clínico Universitario Virgen de la Victoria, Campus de Teatinos s/n, 29010, Málaga Spain; 3 Sanofi, batiment Claude Bernard, 13 quai Jules Guesde, BP14, 94403 Vitry-sur-Seine Cedex France; 4 Sanofi Oncology, 55 Cambridge Parkway, Cambridge, Massachusetts 02142, USA; 5 Institut de Cancérologie de l’Ouest - René Gauducheau, Boulevard Professeur Jacques Monod, 44805 Saint-Herblain-Nantes Cedex, France

**Keywords:** Phase I, Cabazitaxel, Solid tumour

## Abstract

**Background:**

Cabazitaxel is approved in patients with metastatic hormone-refractory prostate cancer previously treated with a docetaxel-containing regimen. This study evaluated a weekly cabazitaxel dosing regimen. Primary objectives were to report dose-limiting toxicities (DLTs) and to determine the maximum tolerated dose (MTD). Efficacy, safety and pharmacokinetics were secondary objectives.

**Methods:**

Cabazitaxel was administered weekly (1-hour intravenous infusion at 1.5–12 mg/m2 doses) for the first 4 weeks of a 5-week cycle in patients with solid tumours. Monitoring of DLTs was used to determine the MTD and the recommended weekly dose.

**Results:**

Thirty-one patients were enrolled. Two of six patients experienced DLTs at 12 mg/m^2^, which was declared the MTD. Gastrointestinal disorders were the most common adverse event. Eight patients developed neutropenia (three ≥ Grade 3); one occurrence of febrile neutropenia was reported. There were two partial responses (in breast cancer) and 13 patients had stable disease (median duration of 3.3 months). Increases in C_max_ and AUC_0–t_ were dose proportional for the 6–12 mg/m^2^ doses.

**Conclusion:**

The MTD of weekly cabazitaxel was 12 mg/m^2^ and the recommended weekly dose was 10 mg/m^2^. The observed safety profile and antitumour activity of cabazitaxel were consistent with those observed with other taxanes in similar dosing regimens.

**Trial registration:**

The study was registered with ClinicalTrials.gov as NCT01755390.

## Background

Taxanes (including docetaxel and paclitaxel) are used extensively in the treatment of solid malignancies [1]. Although taxane-based therapy is associated with favourable antitumour activity, constitutive and acquired resistance limits its use [2]. Therefore, novel taxane-based therapies that demonstrate antitumour activity in taxane-resistant cancers are needed to extend patient survival.

Cabazitaxel is a second-generation microtubule-stabilising taxane that binds to tubulin and has a broad spectrum of antitumour activity, including tumours poorly sensitive, or not sensitive, to docetaxel [3].

Preclinical studies have demonstrated that cabazitaxel has comparable activity to docetaxel in an array of docetaxel-sensitive cell lines, and efficacy in docetaxel-resistant cell lines [3]. Preclinical studies have also shown that cabazitaxel has antitumour activity against a large spectrum of murine and human tumours [4-6]. Furthermore, in animal models, cabazitaxel was able to cross the blood–brain barrier, as evidenced by efficacy against intracranial xenografts [3,7]. Promising results in preclinical studies were confirmed in early-phase clinical studies that demonstrated that cabazitaxel has a manageable toxicity profile as well as antitumour activity in a range of solid tumours, including those progressing under taxane-based therapy [8-10]. In addition, results from the pivotal Phase III trial (TROPIC; NCT00417079) demonstrated that, compared with mitoxantrone, cabazitaxel improved survival in patients with metastatic castration-resistant prostate cancer (mCRPC) who had previously received docetaxel [11]. Cabazitaxel (25 mg/m^2^ once every 3 weeks) plus prednisone has been approved by the US Food and Drug Administration (FDA) and the European Medicines Agency (EMA), as well as by numerous health authorities worldwide for the treatment of men with hormone refractory metastatic prostate cancer (more recently known as mCRPC) previously treated with a docetaxel-containing regimen [12,13]. Additional dosing schedules and treatment combination regimens with cabazitaxel in a variety of tumours are also being investigated.

The primary objectives of this study were to determine the maximum tolerated dose (MTD) and dose-limiting toxicities (DLTs) of cabazitaxel when given as a weekly 1-hour intravenous (IV) infusion for the first four consecutive weeks of a 5-week treatment cycle. Secondary objectives were to establish the safety and pharmacokinetic (PK) profiles of cabazitaxel, to evaluate efficacy and to inform the recommended dose and time interval for future development.

## Methods

### Eligibility and patient characteristics

The study included patients (18–70 years of age) with progressive, histologically proven, advanced neoplastic disease that was either refractory to conventional treatment or for which no standard therapy existed. Patients were required to have adequate organ function, a life expectancy of ≥ 12 weeks and Eastern Cooperative Oncology Group performance status 0–2. Prior therapies were permitted providing they had been terminated a minimum of 4 weeks prior to the start of the study (depending on the therapy), and the patient had fully recovered from any toxic effects of treatment. Prior paclitaxel and/or docetaxel treatment was permitted providing there was no residual evidence of taxane toxicity (exceptions included alopecia [any grade] or peripheral neuropathy [not exceeding Grade 1]).

Patients with haematological malignancies or symptomatic brain metastases were ineligible, as were pregnant or lactating women and those receiving concurrent treatment with other experimental drugs, any anticancer therapy or corticosteroids (unless as chronic treatment). Patients were also ineligible if they had another serious medical condition, current peripheral neuropathy of any origin or a history of severe allergic reaction to docetaxel or paclitaxel. Patients who had received previous extensive radiotherapy (> 20% of bone marrow area), prior intensive therapy with autologous stem cell rescue, more than two prior chemotherapy regimens for advanced disease or more than two regimens containing mitomycin C or nitrosoureas were also excluded from the study.

The study was conducted according to the Declaration of Helsinki (Somerset West Amendment, October 1996) and in compliance with all local regulations. Written informed consent was obtained from patients prior to the initiation of any protocol-specific procedure. The protocol was approved by two independent Ethics Committees; Comité Consultatif de protection des personnes dans la Recherche, Biomedical No. 2, Cellule de Promotion, Immeuble Deurbroucq, 5 allée de l’Île Gloriette, 44093 Nantes, France (Chairperson Dr D. Duveau) and Comité Ético de Investigación Clínica, Ciudad sanitaria Vall d‘Hebrón, 08035 Barcelona, Spain (Chairperson Dr J.B. Montoro-Ronsano).

### Study design

The study was a two-centre, open-label, single-arm, dose-finding study of cabazitaxel given as a weekly 1-hour IV infusion to adult patients with advanced solid tumours. The dose escalation protocol was based on the Simon 4A design [14] without intrapatient dose escalation.

Toxicity was graded according to The National Cancer Institute Common Toxicity Criteria (NCI-CTC, version 2.0) [15]. Non-dose-limiting toxicities (non-DLTs) were defined as Grade 2–4 haematological toxicity (excluding neutropenia Grade 4 for > 5 days, Grade 4 neutropenia with fever for ≥ 1 day or Grade 4 thrombocytopenia) or Grade 2 non-haematological toxicity (excluding alopecia, nausea, vomiting and hypersensitivity). DLTs were defined as Grade 4 neutropenia (absolute neutrophil count < 0.5 × 10^9^/l) lasting more than 5 days, Grade 4 thrombocytopenia (platelet count < 10 × 10^9^/l), febrile neutropenia (Grade 4 neutropenia with fever ≥ 38.5°C [single elevation in oral temperature]) or Grade 3 or 4 non-haematological toxicity (excluding alopecia, nausea, vomiting and hypersensitivity).

### Selection of doses in the study

A dose of 1.5 mg/m^2^, which corresponds to one-tenth of the severely toxic dose in 10% of mice, was chosen as the starting dose. Eight dose levels were planned (1.5, 2.1, 3, 4.2, 6, 8.4, 10 and 12 mg/m^2^). Of note, the 10 mg/m^2^ dose level was added as a protocol amendment. At the first cycle, one patient per dose level was to be included until a non-DLT or DLT was observed. If the patient did not experience a non-DLT or DLT, a double-step (100%) dose increment was allowed. If any patient experienced a non-DLT at the first cycle, at least three patients were to be included at the dose level at which it occurred, with a single-step (40%) dose increment permitted if no DLT occurred among these three patients. If any patient exhibited a DLT at a given dose level, a total of at least six patients had to be included at that dose level. A single dose reduction was only to be made in the case of a DLT. The replacement of patients who were not eligible or evaluable was permitted during the first cycle. Patients who had withdrawn from the study were not re-included.

The MTD was defined as the dose at which two or more of three or six patients developed a DLT in the first cycle of therapy. The recommended dose was defined as one dose level below the MTD.

### Drug administration

Patients were hospitalised for 24 hours during their first cabazitaxel administration. Blood pressure and heart rate were monitored immediately before, during and after administration. Cabazitaxel was administered as a 1-hour IV infusion on Days 1, 8, 15 and 22 of each 5-week cycle until there was evidence of disease progression, unacceptable toxicity or the patient withdrew. No therapy was administered in the last week of the treatment cycle. Premedication was not required in this study.

### Safety evaluation

Patients were evaluable for safety if they started at least one infusion of cabazitaxel and were not lost to follow-up after the first infusion.

All enrolled patients underwent a complete health evaluation at least 7 days before treatment commenced. On the day of cabazitaxel administration, patients were evaluated to ensure baseline characteristics were within safe limits (absolute neutrophil count ≥ 1.0 × 10^9^/l; platelet count ≥ 75 × 10^9^/l; recovery of all prior severe [Grade 3–4] non-haematological toxicities; alanine aminotransferase/alkaline phosphatase/aspartate aminotransferase ≤ Grade 1; and total bilirubin/serum creatinine within normal limits). The duration of a cycle was 5 weeks, but the interval between two cycles could be extended to 6 weeks (i.e. 1 week delay between cycles). If such a delay occurred, patients were required to demonstrate absolute neutrophil count ≥ 1.5 × 10^9^/l and platelet count ≥ 100 × 10^9^/l, in addition to the above parameters, prior to reinitiation of treatment. If recovery from the previous cycle did not occur within 3 weeks, patients were removed from the study. Additionally, during therapy, neurological, toxicity and general health checks were performed every week, and a cardiology examination (electrocardiogram, with Holter scan if patients showed evidence of clinical dysrythmias) was conducted before the start of the trial, every 5 weeks thereafter, and again 3–4 weeks after the last infusion of study drug. Biochemistry monitoring was performed on Days 1, 3, 7, 14, 21 and 28. All safety evaluations were repeated 3–4 weeks after the last infusion to identify late-onset adverse events.

Concomitant medication was allowed in the case of anaphylactic or hypersensitivity reaction, fluid retention or diarrhoea. Furthermore, curative use of colony-stimulating factors was permitted provided that patients demonstrated clinical evidence of neutropenic infection.

### Efficacy evaluation

An assessment of efficacy (according to RECIST criteria) was conducted in patients who received at least one treatment cycle, had at least one follow-up tumour assessment and had no major on-study protocol deviations. Efficacy endpoints were best overall response (defined as the rate of complete and partial response, recorded from the start of treatment until disease progression), duration of response, time to progression and overall survival.

Response was assessed via independently reviewed X-ray, ultrasound and computed tomography scans of all uni- or bi-dimensionally measurable lesions. The presence of measurable lesions was not a requirement of the study.

### Pharmacokinetic analysis

Blood samples (3 ml) were collected from all patients for assessments during cycles 1 and 2. Sampling was performed on Days 1 and 22 at 5, 15, 30 and 60 minutes and 2, 4, 6, 10, 24, 48 and 72 hours post-infusion. In addition, samples were taken immediately prior to infusion and at 30 and 55 minutes during the infusion on Days 1 and 22 (cycle 1). Cerebrospinal fluid (CSF; 1.5 ml) and plasma samples (3 ml) were collected 15 minutes post-infusion when possible. Cabazitaxel levels in plasma and CSF were measured using a validated liquid chromatography/tandem mass spectrometry method [16].

Accuracy, defined as the percentage difference between the nominal and the mean measured concentrations of quality controls, ranged from −4.1% (n = 74) to 6.4% (n = 75) during the analysis period. The precision of the assay, established by the coefficients of variation (CVs) of the quality controls, ranged from 9.9% to 14% during the analysis period, and the accuracy of the dilution controls (1:2 or 1:4) was −4.2% (n = 18), with an associated precision of 3.4%.

PK analysis was carried out using WinNonlin software, version 3.3 (Pharsight, USA). The calculation of PK parameters was performed by a non-compartmental analysis. The maximum observed plasma concentration (C_max_) and area under the plasma concentration–time curve (AUC) from time 0 to the time of the last measurable concentration (AUC_0–t_) were determined. Accumulation ratios were estimated at cycles 1 and 2 (C_max_ Day 22/C_max_ Day 1 and AUC_0–t_ Day 22/AUC_0–t_ Day 1).

### Statistical methods

Statistics were analysed using UNIX® with SAS® software, Version 6.12 (SAS Institute, USA). Descriptive statistics were provided by initial planned dose (safety- and efficacy-evaluable patients) and overall dose level (safety only). Furthermore, safety analyses were performed by patient (worst grade under treatment or during follow-up), by cycle (worst grade or acute adverse events) and at first cycle (worst grade). Kaplan–Meier estimates were performed to analyse censored data in the efficacy population.

Descriptive statistics were reported for each PK parameter by dose level; the dose proportionality of the exposure was assessed on AUC_0–t_ and C_max_ after dose normalisation using the Proc GLM procedure of SAS software (Version 8.2; SAS Institute, USA). A test of linearity was applied on the parameters C_max_ and AUC_0–t_ against the dose expressed in mg/m^2^ followed by the Proc REG procedure of SAS software. The effect of the day (Day 22 versus Day 1) or the cycle (cycle 2 versus cycle 1) on the exposure (C_max_ and AUC_0-t_) after dose normalisation and log transformation was assessed using a Proc MIXED procedure of SAS software with the patient taken as random effect and with day and cycle as fixed effects.

## Results

### Patient characteristics

Thirty-one patients were enrolled; all were evaluable for safety, and 27 were evaluable for tumour response. All patients who received at least one administration of cabazitaxel were included in these analyses. Baseline patient and disease characteristics are summarised in Table 1.

**Table 1:** Patient baseline characteristics

	**Planned dose level, mg/m**^**2**^						
	**1.5**	**3**	**6**	**8.4**	**10**	**12**	**Total**
	**(n = 1)**	**(n = 1)**	**(n = 4)**	**(n = 12)**	**(n = 7)**	**(n = 6)**	**(N = 31)**
Age, years, median (range)	–	–	48	52	44	55	**51**
	(67–67)	(36–36)	(39–51)	(35–69)	(31–65)	(34–70)	**(31–70)**
Male, n (%)	1 (100)	0	1 (25.0)	2 (16.7)	3 (42.9)	2 (33.3)	**9 (29.0)**
Caucasian, n (%)	1 (100)	1 (100)	4 (100)	12 (100)	7 (100)	6 (100)	**31 (100)**
ECOG performance status, n (%)							
0	1 (100)	–	1 (25.0)	2 (16.7)	4 (57.1)	4 (66.7)	**12 (38.7)**
1	–	–	3 (75.0)	9 (75.0)	2 (28.6)	2 (33.3)	**16 (51.6)**
2	–	1 (100)	–	1 (8.3)	1 (14.3)	–	**3 (9.7)**
Tumour type, n (%)							
Breast	NA	NA	NA	NA	NA	NA	**13 (41.9)**
Ovary	NA	NA	NA	NA	NA	NA	**3 (9.7)**
Stomach	NA	NA	NA	NA	NA	NA	**3 (9.7)**
Small bowel	NA	NA	NA	NA	NA	NA	**2 (6.5)**
Other	NA	NA	NA	NA	NA	NA	**10 (32.3)**
Prior anticancer therapy, n (%)							
Chemotherapy only	–	–	–	1 (8.3)	–	–	**1 (3.2)**
Immunotherapy*	–	–	–	–	1 (14.3)	–	**1 (3.2)**
Surgery only	1 (100)	–	–	–	–	–	**1 (3.2)**
Combination therapy	–	1 (100)	4 (100)	11 (91.7)	6 (85.7)	6 (100)	**28 (90.3)**

* Immunotherapy + biological markers. *NA* not available.

### Treatment exposure, delays and dose reductions

The median number of cycles (all dose levels combined) was two (range: 1–9 cycles) and the combined median relative dose intensity was 100%. Median relative dose intensity was not reported for the 1.5 and 3 mg/m^2^ dose levels, because only one and two patients were enrolled, respectively. The median relative dose intensity for the remaining dose levels was 99%, 100%, 100% and 91% for 6, 8.4, 10 and 12 mg/m^2^, respectively. With all dose levels included, median relative dose intensity was 100%. The median cumulative dose was 46.7 mg/m^2^ (range: 6.0–297.8 mg/m^2^). Four cycles were administered at a reduced dose due to non-haematological toxicities (8.4 and 12 mg/m^2^ dose level).

### Dose-limiting toxicities and maximum tolerated dose

No patients at the 1.5, 3, 6 or 8.4 mg/m^2^ dose levels experienced any DLT at the first cycle. Two of the six patients who received the 12 mg/m^2^ dose experienced DLTs (Table 2) (both Grade 3 diarrhoea, including one serious event leading to study discontinuation); as a result, the MTD was reached at 12 mg/m^2^. As per the original protocol, 8.4 mg/m^2^ was initially deemed the recommended IV dose. However, the protocol was subsequently amended to include a dose level of 10 mg/m^2^, an intermediate dose level between the recommended IV dose of 8.4 mg/m^2^ and the MTD of 12 mg/m^2^. At the point of the amendment, 11 patients had already been enrolled and treated at the 8.4 mg/m^2^ dose level; five patients in the dose-escalation phase (three as per protocol, plus two who were waiting for the next dose level to be released) and a further six patients to confirm 8.4 mg/m^2^ as the recommended dose. At the end of the study, a patient who had been planned to receive the 10 mg/m^2^ dose was enrolled at the 8.4 mg/m^2^ dose. This information accounts for the greater number of patients in the 8.4 mg/m^2^ group compared with the 10 mg/m^2^ group.

**Table 2:** Dose-limiting toxicities at cycle 1 and adverse events at subsequent cycles

**DLTs at the first cycle (n = 3)**		
**Dose level, mg/m**^**2**^	**Patients, n**	**Event**
8.4	–	–
10	1	Grade 3 diarrhoea
12	2	Grade 3 diarrhoea (both patients)
**Adverse events at cycles ≥ 2 (n = 13)**		
**Dose level, mg/m**^**2**^	**Patients, n**	**Event**
8.4	4	Grade 3 asthenia (three patients)
		Grade 3 diarrhoea
10	1	Grade 3 haematuria
		Grade 3 dysuria
12	3	Grade 3 asthenia (two patients)
		Febrile neutropenia
		Neutropenia Grade 4 for > 5 days (two patients)
		Grade 3 diarrhoea
		Grade 3 asthenia

One of the seven patients treated with the 10 mg/m^2^ dose experienced a DLT (Grade 3 diarrhoea). Therefore, 10 mg/m^2^ was defined as the revised recommended IV (weekly) dose for further clinical investigations.

### Safety

The most frequently reported non-haematological adverse events regardless of relationship to study drug included pain (97%, most of which was tumour-related [77.4%]), gastrointestinal toxicities (81%, mostly diarrhoea [52%]), fatigue (68%), sensory neuropathy (36%, of which 26% was Grade 1 and 10% was Grade 2), alopecia (19%) and peripheral oedema (16%). Severe liver toxicities were reported in two patients (one patient with Grade 3 alkaline phosphatase and Grade 3 gammaglutamyl transferase at cycle 1 and one patient with Grade 3 alkaline phosphatase at cycle 3); both patients withdrew from the study due to progressive disease. Overall, neutropenia was reported in eight patients. Two patients experienced Grade 4 neutropenia (both received 12 mg/m^2^) and one patient experienced Grade 3 neutropenia (8.4 mg/m^2^ dose level). Febrile neutropenia was observed in one patient (Table 3). The median time to neutrophil nadir was 22 days (range: 17–32 days) and 74% of patients completed their treatment without neutropenic complications. Neutrophil nadir was not dose dependent. Anaemia was frequent but only one patient developed Grade 4 toxicity at 8.4 mg/m^2^.

**Table 3:** Adverse events related to the study drug (all cycles)

	**Planned dose level, mg/m**^**2**^												**All**	
													**(N = 31)**	
	**1.5**		**3**		**6**		**8.4**		**10**		**12**			
	**(n = 1)**		**(n = 1)**		**(n = 4)**		**(n = 12)**		**(n = 7)**		**(n = 6)**			
**Adverse event, n**	G3	G4	G3	G4	G3	G4	G3	G4	G3	G4	G3	G4	**G3**	**G4**
**Non-haematological**														
Fatigue	–	–	–	–	–	–	3	–	–	–	2	–	**5**	**–**
Diarrhoea	–	–	–	–	–	–	1	–	1	–	3	–	**5**	**–**
Haematuria	–	–	–	–	–	–	–	–	1	–	–	–	**1**	**–**
Febrile neutropenia	–	–	–	–	–	–	–	–	–	–	1	–	**1**	**–**
Dysuria	–	–	–	–	–	–	–	–	1	–	–	–	**1**	**–**
**Haematological**														
Neutropenia	–	–	–	–	–	–	1	–	–	–	–	2	**1**	**2**
Anaemia	–	–	–	–	–	–	–	1	–	–	–	–	**–**	**1**
**Days to neutrophil nadir, median (range)**	–		–		–		17		–		–		**22**	
	–		32–32		31–31		17–22		18–18		21–22		**17–32**	
**Median neutrophil nadir, x 10**^**9**^**/l (range)**	–		–		2.3		2.2		2.2		3.1		**2.3**	
	(3.4–3.4)		(1.8–1.8)		(1.4–9.0)		(0.9–5.0)		(1.2–5.7)		(0.2–7.3)		**(0.2–9.0)**	

*G3* Grade 3, *G4* Grade 4.

Eight patients experienced adverse events possibly related to the study drug. The most frequently reported included fatigue (45%), diarrhoea (39%) and nausea (32%).

Five serious adverse events (diarrhoea, dysuria/haematuria, febrile neutropenia and peripheral oedema) were reported in four patients; all occurred at the 10 and 12 mg/m^2^ dose levels. Eight out of 31 patients (26%) were withdrawn from the study because of adverse events, although in two of these patients the adverse events were probably not related to the study drug. Fifteen deaths were reported and progressive disease was the cause of all but one death, which resulted from pneumonia that was not deemed to be related to cabazitaxel administration.

### Efficacy

Of the 31 patients treated, 27 were evaluable for response. There were two confirmed partial responses at 8.4 and 12 mg/m^2^; both patients had breast cancer. A third patient had an unconfirmed partial response. In addition, 13 patients had stable disease (median duration of 3.3 months) and 11 had progressive disease (Table 4). The duration of the two partial responses was 5+ and 2+ months (8.4 and 12 mg/m^2^, respectively) from first infusion to censored date. The median time to progression was 2.1 months (95% confidence interval 0.99–3.22; range: 0–10.4 months), although six patients were censored (five for further antitumour therapy and one lost during follow-up). Median overall survival was 15.6 months (range: 0.99–32.7 months).

**Table 4:** Doses that yielded the best overall responses

	**Best overall response**^**a**^		
**Planned dose level, mg/m**^**2**^	**Investigator’s response**	**Response review meeting (expert)**	**Final evaluation**
**8.4**	PR	PR	**PR**
	PR	NC	**NC**
**10**	NC^b^	NC^c^	**NC**^**c**^
**12**	PR	PR	**PR**

^a^ Each response represents one patient. ^b^ CR at cycle 2. ^c^ PR at cycle 2.

*CR* complete response, *PR* partial response, *NC* no change.

### Pharmacokinetics

**Figure 1:**
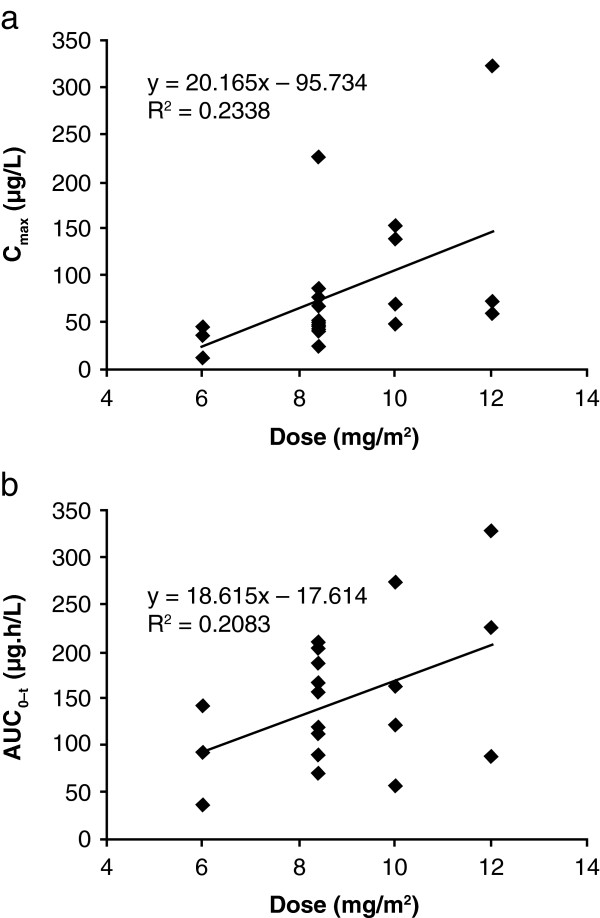
**Cabazitaxel pharmacokinetics. (a)** C_max_ versus dose and **(b)** AUC_0–t_ versus dose at the first administration at cycle 1 (n = 21).

PK evaluation was carried out in 31 patients during the first two cycles. After the first administration of 10 mg/m^2^ in cycle 1, cabazitaxel was detectable up to 6, 10, 23 and 73 hours after the end of infusion in the four patients for whom PK evaluation was possible. After the fourth infusion, cabazitaxel was detectable up to 10 hours (one patient), 48 hours (one patient) and 72–74 hours (two patients) after the end of infusion in the four patients for whom PK evaluation was possible. The PK parameters of cabazitaxel are shown in Table 5. The increase in C_max_ and AUC_0–t_ after the first administration in cycle 1 appeared dose-proportional from 1.5–12 mg/m^2^. Following exclusion of the patients treated at 1.5 and 3 mg/m^2^ (n = 1 each), statistical analysis confirmed that C_max_ and AUC_0–t_ were dose-proportional from 6–12 mg/m^2^ (Figure 1). There was a significant increase (41%; p = 0.0016) in AUC_0–t_ on Day 22 compared with Day 1 (both cycle 1). However, C_max_ remained unchanged compared with Day 1. Furthermore, there were no statistical differences in C_max_ and AUC_0–t_ between cycles 1 and 2 on either Days 1 or 22. Cabazitaxel was not detected (limit of quantification, 1 μg/l) in four samples of CSF collected up to 35 minutes after the end of infusion in three patients receiving 3 mg/m^2^ and 6 mg/m^2^ doses. Low (≤ 14.4 μg/l) concomitant plasma concentrations of cabazitaxel were reported in two patients (one taken 3 minutes and one taken 5 minutes prior to lumbar puncture).

**Table 5:** Pharmacokinetic parameters of cabazitaxel on Days 1 and 22 (cycle 1)

	**Day 1**			**Day 22**		
**Dose, mg/m**^**2**^	**Patients treated/with data**	**C**_**max,**_^**a **^**μg/l**	**AUC**_**0–t**_**, μg.h/l**	**Patients treated/with data**	**C**_**max,**_^**a **^**μg/l**	**AUC**_**0–t**_**, μg.h/l**
1.5	1/1	15.5	17.2	1/1	13.5	28.2
3	1/1	41.9	47.1	1/1	23.0	102
6	4/3	31.2 ± 15.5 (50)	89.7 ± 51.8 (58)	4/3	49.4 ± 13.8 (28)	150 ± 34 (22)
8.4	12/11	71.7 ± 54.4 (76)	144 ± 46 (32)	11/9	70 ± 30.4 (43)	190 ± 89 (47)
10	7/4	102 ± 52 (51)	153 ± 91 (60)	4/4	89.3 ± 80.6 (90)	187 ± 56 (30)
12	6/3	152 ± 148 (97)	212 ± 120 (56)	4/2	84.8–480	145–447

Data are expressed as mean ± SD (CV%).

^a^ Observed value at 5 minutes before the end of infusion except for four patients on Days 1 and for seven patients on Day 22 for whom maximal plasma concentrations were measured 20 minutes before the end of infusion.

## Discussion

Cabazitaxel is a second-generation taxane that has demonstrated efficacy against taxane-resistant tumours in both *in vitro* and *in vivo* models [3-6,9-11,17]. Preclinical studies reported that intermittent dosing schedules (vs split-dose schedules) allowed the highest dose of cabazitaxel to be administered with the best patient recovery and optimum antitumour activity [18]. In addition, previous animal studies indicated that the efficacy and toxicity of cabazitaxel are schedule-dependent [5]. Therefore, this study was designed to assess DLTs and the MTD (and thus the recommended dose) of a weekly dosing regimen, with patients receiving cabazitaxel weekly for the first 4 weeks of a 5-week treatment cycle. These results provided the basis for the 5-week treatment cycle described here. Thirty-one patients were enrolled in this Phase I dose-finding study. Investigation of the PK, and safety profiles and efficacy of cabazitaxel were secondary objectives.

The MTD for weekly administration without premedication was established as 12 mg/m^2^; 10 mg/m^2^ was the dose recommended for future clinical studies using a weekly dosing regimen. There have been several studies investigating the use of cabazitaxel in solid tumours, mostly with a 3-weekly administration schedule [8-11]. The results of these studies, conducted contemporaneously with the current study, provide some evidence that a 3-weekly dosing schedule may be associated with more favourable toxicity and efficacy profiles compared with a weekly regimen. They also reported different MTD and recommended doses; Mita and colleagues [9] explored the use of cabazitaxel in advanced solid tumours and recommended a 20 mg/m^2^ dose, whereas a similarly designed Phase I study recommended a 25 mg/m^2^ dose for future studies [8]. Further evaluation of 3-weekly dosing was carried out in a Phase II study in patients with metastatic breast cancer, in which patients received 20 mg/m^2^, with escalation to 25 mg/m^2^ if no significant adverse event was observed at cycle 1 [10]. The study reported promising efficacy results, with two complete and eight partial responses. The results from the 3-weekly Phase I and Phase II trials informed the design of the Phase III TROPIC (NCT00417079) trial of cabazitaxel in combination with prednisone in patients with mCRPC previously treated with a docetaxel-containing regimen [8,9]. This pivotal trial was the basis for the approval by the FDA and EMA of cabazitaxel in combination with prednisone in that patient population [12,13]. A trial comparing 20 mg/m^2^ and 25 mg/m^2^ doses of cabazitaxel in a head-to-head study in patients with mCRPC is ongoing.

In this study, the median relative dose intensity was 100% across all dose levels, resulting in an absolute. The dose intensity of 10 mg/m^2^/week (3-weekly regimen) was one of several dose levels assessed in a previous Phase I; median relative dose intensity was similar to that reported here [9]. Furthermore, in the pivotal Phase III TROPIC study, in which the approved regimen (25 mg/m^2^ once every 3 weeks) was used, the absolute dose intensity was slightly lower, at 8.3 mg/m^2^/week (relative dose intensity 96.1%) compared with the results shown here with a lower dose [11].

In the current study, cabazitaxel given on a weekly schedule appeared to be tolerable with a manageable side-effect profile, despite the fact that cabazitaxel was administered without premedication. Notable toxicities reported in this study included both neutropenia and diarrhoea, which are consistent and predictable side effects of taxane-based therapy. The most common Grade ≥ 3 toxicities were fatigue and diarrhoea. The toxicity results presented here are similar to, but with lower incidence rates than, those reported in the TROPIC trial, where cabazitaxel was administered under a different schedule [11]. Analyses of results from the TROPIC trial demonstrated that these adverse events can be appropriately managed with proactive treatment and conscientious monitoring. Specifically, prophylactic use of granulocyte colony-stimulating factor reduced the severity of neutropenia in patients receiving cabazitaxel plus prednisone [19], and diarrhoea was resolved with supportive treatment.

The efficacy results presented from the current Phase I study are consistent with those observed in other cabazitaxel studies with other schedules of administration, demonstrating the activity of the agent against a variety of solid tumours in patients who have received prior anticancer therapy [9,10]. Indeed, in this study, over half of the patients experienced either stabilised or improved responses, despite the advanced nature of their disease. Furthermore, in the dose-escalation part of the study, treatment with cabazitaxel resulted in median overall survival of 15.6 months and a median time to progression of 2.1 months. It is notable that Phase III studies with docetaxel in prostate cancer have demonstrated that a weekly regimen may be associated with inferior efficacy to a 3-weekly regimen [20]. Based on these data, it is unlikely that a weekly regimen of cabazitaxel in patients with prostate cancer will progress to Phase III trials.

Available data indicate that cabazitaxel PK appears to be dose proportional. Population analyses carried out on pooled PK data, from Phase I, II and III studies, including cabazitaxel plasma concentrations obtained after the first administration in patients treated at 10 or 12 mg/m^2^ from this study, facilitated the estimation of half-lives, clearance (CL) and volume of distribution at steady state (V_ss_). PK parameters from this study generated in the pooled analysis (mean CL: 25.5 l/h/m^2^; CV: 36%, mean elimination half-life: 130 hours; CV: 72% and mean V_ss_; 3160 l/m^2^; CV: 66%) were in the range of those estimated in patients with solid tumours or with mCRPC treated using the 3-weekly schedule [16], indicating no marked difference between weekly and 3-weekly regimens.

Consistent with the long elimination half-life, some accumulation in the deep distribution compartment appeared to occur after the fourth administration (within cycle 1) of cabazitaxel, with a significant increase in exposure (AUC_0-t_). However, no difference in exposure was observed between the first administrations of cycles 1 and 2, suggesting that a 2-week treatment break after 4-weekly administrations can avoid the accumulation of cabazitaxel in the plasma, making a weekly administration regimen feasible. As cabazitaxel is able to cross the blood–brain barrier in animals [3,7], four lumbar punctures were undertaken to evaluate cabazitaxel penetration in this study; cabazitaxel was not detected in any CSF samples. However, the negative results do not conclusively demonstrate that cabazitaxel does not enter the CSF, as levels in plasma samples taken within 5 minutes of lumbar puncture were low (≤ 14.4 ng/ml), all four patients had received low doses of cabazitaxel (3–6 mg/m^2^) and a prior study has demonstrated low (8%) free fraction of cabazitaxel in the plasma [8].

Taken together, the results presented here suggest that weekly dosing at the recommended dose of 10 mg/m^2^ may be a feasible treatment regimen in patients with solid tumours. While this regimen is not actively being pursued at this time, other studies evaluating the efficacy of different cabazitaxel doses, alone or in combination with other anticancer agents or radiotherapy, in a range of solid tumours, are currently planned or ongoing.

## Conclusions

The MTD of weekly cabazitaxel was 12 mg/m^2^ and the recommended dose for weekly cabazitaxel was 10 mg/m^2^. The adverse events observed were consistent with other taxanes in similar dosing regimens. Antitumour activity was also comparable with other taxanes in similar dosing regimens. PK results showed that C_max_ and AUC_0–t_ were dose proportional. In conclusion, this study demonstrates promising results with a cabazitaxel weekly dosing regimen, which may provide the foundation for future studies.

## Competing interests

P. Fumoleau is IDMC Chairman for Johnson & Johnson and has acted as a consultant/advisory board member for Abbott, GlaxoSmithKline, Roche and Sanofi. D. Sémiond and S. Gupta are employees of Sanofi and have stock ownership in Sanofi. M. Campone has received honoraria from Novartis and has a consultant relationship with Novartis and Servier. M. Campone has received research funding from Novartis and Cephalon. This study was sponsored by Sanofi. The authors received editorial support from Dr Melissa Purves of MediTech Media, funded by Sanofi.

## Authors’ contributions

The study was conducted in two centres under the responsibility of PF and Dr José Baselga. PF and Sanofi authors were responsible for conception and design of the study. Study management, monitoring, statistical analysis and PK analysis were performed by Sanofi authors. Data interpretation was performed by Sanofi authors and PF. All authors reviewed and approved the manuscript.

## Authors’ information

Professor Fumoleau was at Centre René Gauducheau, France, when this study took place.

Dr Trigo was at Vall d’Hebron University Hospital, Barcelona, Spain when this study took place.

## Pre-publication history

The pre-publication history for this paper can be accessed here:

http://www.biomedcentral.com/1471-2407/13/460/prepub
